# The Role of Type III Interferons in Hepatitis C Virus Infection and Therapy

**DOI:** 10.1155/2017/7232361

**Published:** 2017-02-01

**Authors:** Janina Bruening, Bettina Weigel, Gisa Gerold

**Affiliations:** Institute for Experimental Virology, Centre for Experimental and Clinical Infection Research (TWINCORE), Hannover, Germany

## Abstract

The human interferon (IFN) response is a key innate immune mechanism to fight virus infection. IFNs are host-encoded secreted proteins, which induce IFN-stimulated genes (ISGs) with antiviral properties. Among the three classes of IFNs, type III IFNs, also called IFN lambdas (IFNLs), are an essential component of the innate immune response to hepatitis C virus (HCV). In particular, human polymorphisms in IFNL gene loci correlate with hepatitis C disease progression and with treatment response. To date, the underlying mechanisms remain mostly elusive; however it seems clear that viral infection of the liver induces IFNL responses. As IFNL receptors show a more restricted tissue expression than receptors for other classes of IFNs, IFNL treatment has reduced side effects compared to the classical type I IFN treatment. In HCV therapy, however, IFNL will likely not play an important role as highly effective direct acting antivirals (DAA) exist. Here, we will review our current knowledge on IFNL gene expression, protein properties, signaling, ISG induction, and its implications on HCV infection and treatment. Finally, we will discuss the lessons learnt from the HCV and IFNL field for virus infections beyond hepatitis C.

## 1. Type III Interferons

### 1.1. Interferon Lambda Genes and Proteins

Interferons (IFN) are innate cytokines, which interfere with virus infections. While type I IFNs were discovered in the 1950s, it was not until 2003 that the first type III IFNs, namely, IFN lambda 1 (IFNL1), lambda 2 (IFNL2), and IFN lambda 3 (IFNL3), were described [1, 2]. The most recent member of the type III IFNs, IFN lambda 4 (IFNL4), was discovered even ten years later [3, 4]. All four IFNLs are encoded on chromosome 9 in the 19q13.13 region. INFLs share their open reading frame structure with the interleukin-10 (IL-10) family of cytokines comprising five exons and four introns [5–7]. Therefore, they are also termed IL-29 (IFNL1), IL-28A (IFNL2), and IL-28B (IFNL3).

**Figure 1:**
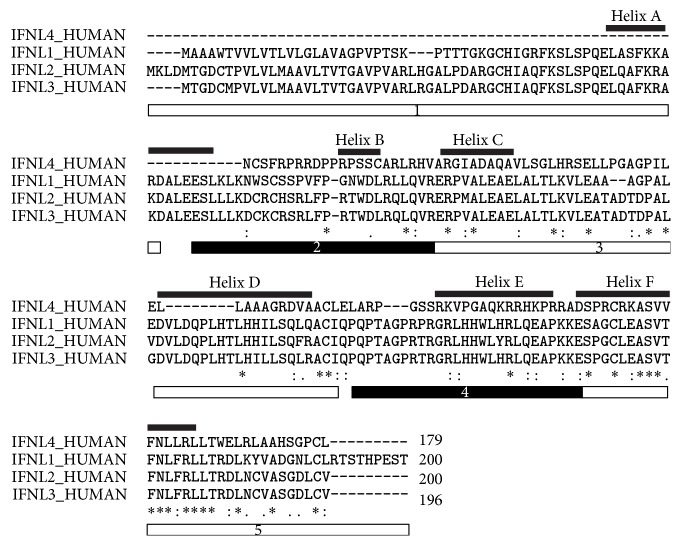
Sequence alignment and amino acid conservation of IFNLs. Clustal Omega (1.2.3) alignment [37] of IFNL proteins (IDs: Q8IU54, Q8IZJ0, Q8IZI9, and K9M1U5). Exons are indicated by the black and white boxes below the sequences. Positions of helices are indicated by the lines above the sequences. Identical amino acids are marked by an asterisk (*∗*); conserved amino acids by a colon (:); and semiconserved amino acids by a period (.).

IFNL1 through IFNL3 have a high degree of sequence similarity with 72% to 96% amino acid conservation with IFNL2 and IFNL3 being most closely related. These findings suggest a common ancestor gene for all IFNLs [3]. IFNL4 expression is the consequence of a frameshift mutation and this gene product shares 27% to 29% sequence similarity with the other three IFNLs (Table 1 and Figure 1). IFNL1–3 proteins are roughly 22 kDa in size, while IFNL4 is slightly smaller with 20 kDa. They share an alpha helical bundle structure with type I and type II IFN family members. Significant differences occur in the side chains of IFNL1, IFNL2, and IFNL3 and amino acid differences at the receptor binding site likely contribute to the differences in IFNL responses as detailed below.

### 1.2. IFNL Expression

The expression of IFNL genes is tightly controlled and expression profiles of IFNL subtypes are ligand and tissue specific [8]. Typically, RNA virus infection and the concomitant exposure of cells to foreign RNA in cytoplasmic or endosomal compartments lead to IFNL induction. In particular, Sindbis virus, dengue virus, vesicular stomatitis virus, encephalomyocarditis virus [1, 2], respiratory syncytial virus [9, 10], influenza virus, Sendai virus [11, 12], and hepatitis C virus (HCV) [13–15] were shown to induce IFNLs in vitro and in vivo. In addition to RNA viruses, DNA viruses including cytomegalovirus and herpes simplex virus can induce IFNLs [16, 17]. While almost any cell type can express IFNLs, the most prominent producers of these antiviral cytokines are myeloid and plasmacytoid dendritic cells [8, 18–21]. Tissues with strong IFNL induction upon virus infection are the lung and the liver with a strong contribution of airway epithelial cells and hepatocytes [15, 22–29]. Limited data is available on the expression kinetics of IFNLs in different cell types. It seems, however, that IFNL expression onset and duration differ for the four subtypes. For instance, primary human hepatocytes (PHH) carrying the single nucleotide polymorphism (SNP) responsible for IFNL4 expression show an early and short IFNL4 expression (2 to 6 h after stimulation), while IFNL3 was detectable from 2 to 24 h after stimulation with a synthetic poly I:C RNA ligand [4]. Differences in positive or negative feedback mechanisms may explain the varying expression kinetics for IFNL subtypes. IFNL1 through IFNL3 are typically induced simultaneously and this is reflected by common transcription factors and binding sites in the promoter regions. Activator protein 1, IFN response factor 3 (IRF3), IRF7, and nuclear factor kappa beta (NF-kB) are thought to bind to the promoter of all INFL genes [11, 12, 30–36]. Additionally, Med23 seems to be a transcriptional coactivator [17]. Taken together, IFNLs are induced upon sensing of virus infection in particular after lung and liver infection.

### 1.3. The IFNL Receptor

The receptor for all four IFNLs is composed of two subunits, the alpha-subunit IFNLR1 encoded on chromosome 1 and the beta-subunit IL10RB encoded on chromosome 21 [40–44]. The former is specific for the IFNL receptor (IFNLR), while the latter is shared with the type II cytokine receptors for IL-10, IL-22, and IL-26 [45]. Restricted expression of the IFNLR1 subunit leads to a tissue specific response to IFNLs. In particular tissues with high epithelial cell content like intestine, liver, and lung express IFNLR1 and respond to IFNLs [46]. Apart from primary human hepatocytes, hepatocellular carcinoma cell lines including Huh-7 and HepG2 cells respond to IFNL [14, 47]. In addition to the full length IFNLR1, a secreted form lacking exon VI has been described and may function as a decoy receptor dampening IFNL responses [2, 48, 49].

The IFNL ligand-receptor interface is comprised of helix A, loop AB, and helix F for IFNL and the N-terminal domain as well as the interdomain hinge region for the IFNLR. Van der Waals and hydrophobic forces determine the ligand-receptor interaction [40]. Amino acids critical for receptor binding differ between IFNL subtypes and this might lead to different ligand binding affinities as well as differences in the stability of the ligand-receptor complex [4, 50]. Additionally, mutations in the IFNL3 and IFNL1 receptor binding sites have been described with the IFNL4 generating frameshift mutation being the best described [40, 41, 50]. The impact of these genetic variants is discussed in detail below. Taken together all four IFNL proteins share the same cell surface receptor, which is primarily expressed in intestine, lung, and liver tissue.

### 1.4. IFNLR Signaling

Signaling in response to IFNLs is initiated by dimerization of the two IFNLR subunits. Initial binding of IFNLs to IFNLR1 induces the recruitment of IL-10RB, leading to the activation of the receptor associated kinases Janus kinase 1 (JAK1) and tyrosine kinase 2 (TYK2). Cross-tyrosine phosphorylation of the IFNLR subsequently recruits signal transducer and activator of transcriptions (STAT) 1 and 2 to the receptor platform. Phosphorylated STAT1 and STAT2 form a heterotrimer together with IFN regulatory factor 9 (IRF9). This trimeric complex, called IFN-stimulated gene factor 3 (ISGF3), translocates to the nucleus where it binds to the IFN regulated response element (ISRE) to drive IFN-stimulated gene (ISG) expression [51].

Antiviral effects of IFNLs are largely shared with type I IFNs. However, differences in receptor tissue expression and the kinetics of STAT pathway induction exist between the two IFN classes [52–54]. In Huh-7 cells, IFNL induces a slower and more sustained ISG response [55, 56]. Among the hundreds of ISGs induced by IFNLs and type I IFNs are ISG15, myxovirus (influenza virus) resistance 1 (MX1), 2′-5′-oligoadenylate synthetase 1–3 (OAS-1–3), and protein kinase R (PKR). ISGs interfere with different stages of viral life cycles as reviewed in [57]. The anti-inflammatory ISGs USP18 and suppressor of cytokine signaling 1–3 (SOCS1–3), however, are specifically induced by IFNLs and not by type I IFNs [58]. Both proteins interfere with STAT signaling and therefore lead to desensitization to type I IFNs and IFNLs [59–61]. IFNL4 additionally induces expression of* rantes* and* fos* genes in hepatoma cells [4]. These genes are hallmarks of HCV-induced liver damage. Interestingly and in contrast to type I INFs, IFNLs are themselves ISGs as IFN stimulation of hepatoma cells induces their expression [11].

Although IFNL2 and IFNL3 have high sequence homology, they differ in their antiviral activity with IFNL3 displaying the strongest antiviral activity in a HepG2 challenge experiment with encephalomyocarditis virus [62]. This finding is in line with a strong ISG (MX1 and IRF9) induction by IFNL3 in hepatocytes [55]. IFNL4, in turn, displays antiviral activities which are comparable to IFNL3 as shown in reporter cells expressing the IFNLR and a luciferase gene under the control of the IFI6 promoter [3]. In conclusion, IFNLs signal through the JAK1/STAT pathway for ISG induction and the set of ISGs largely overlaps with that induced by type I IFNs.

## 2. Hepatitis C Virus

### 2.1. Molecular Virology of HCV

**Figure 2:**
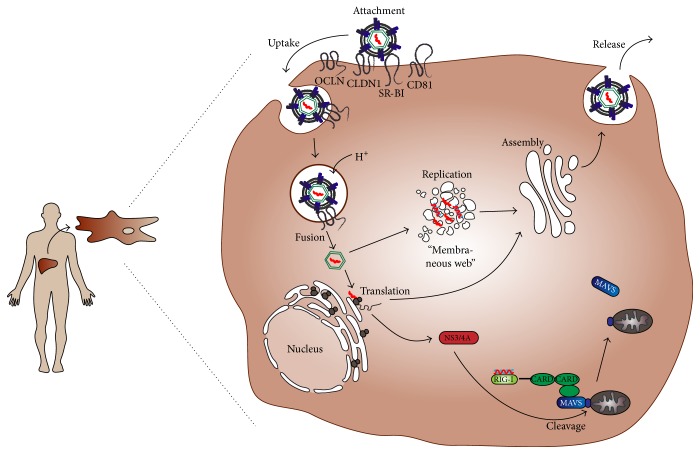
Hepatitis C virus life cycle and MAVS cleavage. Upon transmission, HCV enters hepatocytes in a multistep process involving the four host factors SR-BI, CD81, CLDN1, and OCLN. After uptake and pH-dependent fusion of viral and endosomal membranes, HCV releases its RNA genome into the cytosol of the host cell. Replication via double-stranded RNA intermediates takes place in the membranous web, consisting of ER-derived structures. HCV RNA is then translated into a precursor protein, which is cleaved into ten mature viral structural and nonstructural (NS) proteins. One of the latter, NS3/4A, can proteolytically cleave and by this inactivate MAVS, a RIG-I and MDA5 adaptor protein, which is important for mounting an innate immune response against HCV infection (see text and Figure 3 for details).

The hepatitis C virus belongs to the genus* Hepacivirus* in the Flaviviridae family. HCV is an enveloped virus with a single-stranded, positive-orientated RNA genome of 9.6 kbp length. According to genome sequence diversity HCV can be classified into seven genotypes and multiple subtypes [63]. The liver tropic virus enters hepatocytes in a multistep process involving several host cell proteins (as reviewed, e.g., in [64]). After pH-dependent fusion of the viral membrane with the endosomal membrane, the viral genome is released into the cytoplasm. There the positive-orientated RNA genome is directly translated into a single polyprotein, which is cleaved by viral and cellular proteases into 10 structural and nonstructural (NS) proteins. Replication and virus assembly occurs in endoplasmic reticulum- (ER-) associated membranous structures, called the membranous web (MW) (as reviewed, e.g., in [65]). HCV assembly, maturation, budding, and release occur in close contact with the cellular very low density lipoprotein synthesis pathway. Nascent HCV particles are released from the cells via the secretory pathway into the bloodstream or directly infect bystander cells (as reviewed, e.g., in [64]). A schematic overview of the HCV life cycle is depicted in Figure 2.

### 2.2. Pathogenesis and Treatment of Hepatitis C

Worldwide 92–149 million people, representing approximately 2% of the world's population, are chronically infected with HCV [66], one of the causative agents of viral hepatitis. HCV is a blood borne virus and transmission occurs parenterally, mainly by reusing injection material, insufficient sterilization of medical tools, or by transfusion of unscreened blood or blood products. As screening of blood products is a standard procedure nowadays in most countries, people who inject drugs have the highest risk of contracting hepatitis C. In fact, over 60% of injecting drug users are positive for HCV-antibodies [67].

Naturally HCV infects only humans, but chimpanzees can be experimentally infected. In both cases, HCV targets the liver, in particular hepatocytes. The narrow host range is determined by the presence or absence of certain host cell factors; proteins critically needed for HCV entry, like the cell surface receptors scavenger receptor BI (SRBI), CD81, claudin-1 (CLDN1), and occludin (OCLN) (as reviewed, e.g., in [68]) or molecules needed for viral replication, like microRNA 122, are expressed in hepatocytes. On the other hand, proteins suppressing HCV infection, like EWI-2wint, are absent in hepatocytes [69].

After acute infection, which is mainly asymptomatic, HCV establishes a lifelong, persistent intrahepatic infection in approximately 80% of the patients. Development of chronic hepatitis C (CHC) leads to progressing liver fibrosis and eventually cirrhosis (15–30% of CHC patients), which can cause liver failure or the development of hepatocellular carcinoma (2–4% of CHC induced cirrhosis patients per year) [70]. Consequently, HCV causes app. 700,000 deaths per year [70].

CHC was classically treated with recombinant PEG-IFN-alpha in combination with Ribavirin (RBV). The treatment duration was long (24–48 weeks) and wearing, with PEG-IFN-alpha being administered 3 times a week, severe side effects occurring frequently, and still only approximately half of the patients being cured. Since 2014 HCV therapy improved drastically, as several direct acting antivirals targeting HCV NS3/4A protease, NS5A, or NS5B RNA-polymerase were approved. These inhibitors, either alone or in combination with RBV, now heal over 90% of patients treated. Direct acting antivirals (DAAs) are more effective than PEG-IFN-alpha in eliminating HCV, but also treatment duration is shorter (minimum of 8 weeks), they can be administered orally, and adverse events are fewer. Despite advances in drug development, a vaccine against HCV is still not available. For more details we refer the reader elsewhere, for example [71, 72].

### 2.3. Innate Immunity to HCV

**Figure 3:**
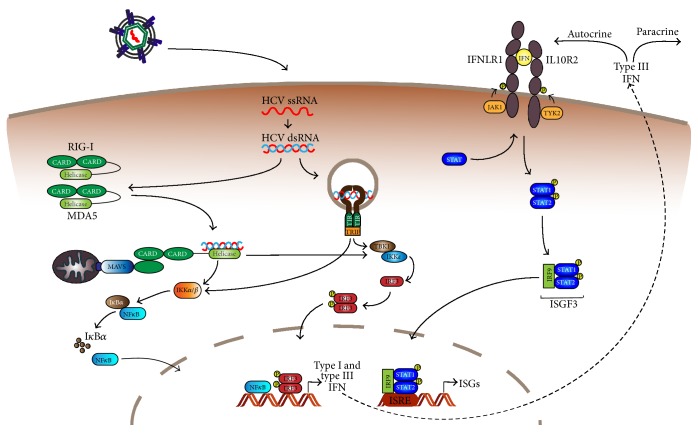
Innate immunity against HCV. In HCV infected cells dsRNA replication intermediates are recognized by the cytosolic RNA sensors RIG-I and MDA5, leading to the activation of MAVS. Endosomal dsRNA is recognized by TLR3, leading to the activation of TRIF. MAVS and TRIF signal via the kinases IKK and TBK1, resulting in translocation of the transcription factors NF-*κ*B and IRF3 to the nucleus. Here they trigger the expression of type I and type III IFNs, which are secreted and bind to their receptors in an auto- or paracrine manner. Subsequently, the JAK/STAT pathway is activated, which ultimately initiates the expression of ISGs, generating an antiviral state.

The innate immune response serves as the first line of defense against infections; pathogen associated molecular patterns (PAMPs) are recognized by extra- or intracellular pattern recognition receptors (PRRs), which triggers signaling cascades leading to the production of cytokines including interferons. The innate immune response to HCV is summarized in Figure 3.

In HCV infected cells, double-stranded (ds) RNA replication intermediates are generated and recognized as PAMPs by the cytosolic RNA sensor retinoic acid-induced gene I (RIG-I) [73] and melanoma differentiation-associated gene 5 (MDA5) [74, 75], both belonging to the family of RIG-I like receptors (RLRs). Sensing of HCV by RIG-I or MDA5 then leads to the oligomerization of the adaptor protein mitochondria antiviral signal (MAVS; also called CARDIF, VISA, IPS-1) into large signaling complexes [76].

Besides the cytosol, HCV dsRNA can also be present in extracellular, ER, or endosomal compartments. Extracellular dsRNA, maybe released from dying cells, can be taken up into uninfected neighboring cells by class A scavenger receptors [77]. After endocytosis, dsRNA is brought to the endosome, where it is bound by Toll-like receptor 3 (TLR3). Alternatively, TLR3 might engage HCV in autophagic vesicles, as HCV replicating cells display an enhanced amount of them [78]. Recognition of dsRNA by TLR3 activates TIR domain-containing adapter-inducing IFN-*β* (TRIF; also called TICAM-1) signaling.

MAVS and TRIF then trigger signaling cascades leading to the activation of different cytosolic kinases (I*κ*B kinases (IKK) and TANK-binding kinase 1 (TBK1)), which in turn induce activation of the key transcription factors NF-*κ*B and IRF3 [79, 80]. Upon activation, these proteins translocate to the nucleus where they bind to promoter elements in type I and type III IFN genes. By this, inflammatory cytokine and IFN expression is initiated. Binding of secreted IFNs to their receptors in an autocrine or paracrine manner leads to the activation of the JAK/STAT pathway, as depicted in Figure 3. Ultimately, this triggers the expression of hundreds of IFN-stimulated genes ISGs, which generate an antiviral state limiting HCV replication.

During CHC, the innate immune response can only control HCV replication but not completely eliminate the virus. This is partially due to viral mechanisms counteracting the immune response: Briefly, HCV NS3/4A serine protease has been shown to inhibit IFR3 phosphorylation [81] by cleaving and inactivating the RIG-I adaptor protein MAVS [82–84] and the TLR3 adaptor protein TRIF [85]. Interestingly, recently discovered members of the genus Hepacivirus infecting nonhuman mammals carry an NS3/4A enzyme capable of cleaving not only their cognate host's MAVS, but also human MAVS [86]. This suggests that all yet studied hepaciviruses can antagonize the human antiviral innate immune response and that there is no barrier to zoonotic transmission at the level of innate immune interference.

## 3. IFN Lambda and HCV Infection

### 3.1. Induction and Role of IFN Lambda in HCV Infection

#### 3.1.1. IFNL in CHC Patients

Humans chronically infected with HCV display increased IFNL expression. Specifically, Dolganiuc et al. showed that IFNL serum levels are higher in CHC patients than in HCV-negative with liver inflammation [87]. The authors observed elevated expression of the IFNLR in liver biopsies from CHC patients. Studying liver biopsies from infected patients also helped to confirm that there is an association between IFNL and ISG expression levels; namely, that IFNL expression leads to elevated ISG induction [88, 89]. In particular, a correlation between the activity of the IFNL4 protein and ISG induction has been discovered. Surprisingly, high IFNL4 and ISG levels negatively impacted the outcome of HCV infection and treatment (see Section 3.2) [90].

#### 3.1.2. IFNL in Experimental Animal Models of Hepatitis C

The host immune response to acute HCV infection has been studied in experimentally infected chimpanzees and in genetically humanized mice. In the livers of chimpanzees a strong host response can be detected, including the induction of type III IFN transcription and ISG expression [88, 91]. Especially IFNL1 mRNA and protein levels are elevated, correlating with ISG expression and viremia. However, there is no link between type III IFN expression in the liver or peripheral organs of infected chimpanzees and the outcome of the acute infection [91]. Consistently, in immunocompetent transgenic mice expressing the human HCV entry factors, HCV infection results in upregulation of several ISGs [92]. This is consistent with the observation that mouse-liver derived cells produce type I and III IFNs when transfected with HCV subgenomic RNA and this leads to abrogation of HCV replication [93]. Of note, current mouse models do not allow chronic HCV infection and since the ban on chimpanzee experimentation there are no immunocompetent animal models to study CHC. Recent efforts on establishing alternative nonhuman primate models for hepatitis C [94, 95] and on using rodent hepaciviruses as surrogate infectious agents to study CHC, might resolve this hurdle in the future [96, 97].

#### 3.1.3. IFNL in In Vitro Models of Hepatitis C

In line with observations made in livers of infected chimpanzees HCV infection induces the expression of IFNL in primary human hepatocytes [88, 91]. Type III IFNs and ISGs are similarly inducted upon HCV infection of primary human fetal liver cells [98, 99]. Here the magnitude of induction differs from donor to donor but correlates with virus replication.

To study different aspects of the HCV life cycle, hepatoma cell lines are frequently used. Similar to HCV infection of primary cells, also the hepatoma cell line HepG2 induces IFNL transcription upon infection [74, 88]. Interestingly, Israelow et al. showed that IFNL induction attenuates HCV replication and that the IFNL response in HepG2-HFL cells stably replicating a HCV subgenome is blunted, probably due to MAVS cleavage by HCV NS3/4A [100]. With regard to IFNL4, Hong et al. found that endogenous IFNL4 transcription is only poorly induced upon stimulation with HCV in different hepatoma cell lines and PHH. Also no or reduced levels of secreted IFNL4 as compared to IFNL3 are detectable upon HCV infection [3]. The partial retention of IFNL4 in the cytoplasm, as observed in HepG2 cells and PHH in a different study, might explain this observation [101].

The clear correlation between IFNL induction and HCV attenuation observed in hepatoma cell lines does not reflect observations in CHC patients for several reasons. First, the complexity of the liver with contributions of Kupffer cells, liver sinusoidal endothelial cells, stellate cells, and infiltrating additional immune cells (reviewed in [102]) is obviously not mimicked by simple cell culture models. Second, transformed cell lines do not necessarily resemble primary hepatocytes. In fact, most hepatoma cell lines that can be infected with HCV do not mount a strong innate immune response [73]. Nevertheless hepatoma cell lines are regularly used to study the effect of exogenously added IFNL on HCV infection as they typically express all components of the IFNLR pathway. IFNL stimulation reduces levels of subgenomic or full length genomic HCV (+)RNA in Huh-7 cells in a dose dependent-manner [49, 103, 104]. These results were confirmed in several other hepatoma cell lines, including the Huh-7 derived Lunet hCD81 cells expressing a firefly luciferase gene or HepG2 cells expressing microRNA122 and CD81 [3].

Hepatoma cell lines and PHH have also been used to study how the IFNL subtypes differ in their ability to limit viral infections; IFNL3 and IFNL4 induce the same set of ISGs in PHH [105] and the two subtypes have the same antiviral activity against HCV in an overexpression setup in hepatoma cells. In summary, expression of specific IFNL subtypes is induced in PHH and some hepatoma cell lines upon infection with HCV, resulting in limiting virus production. However, the majority of hepatoma cell lines do not elicit a strong immune response and IFNL expression. Novel model systems including stem cell derived hepatocytes [106–108] and tissue engineering systems [109] might in the future allow to more faithfully mimic host responses to hepatotropic virus infection.

### 3.2. IFN Lambda Polymorphisms

After establishment of PEG-IFN-alpha and RBV as the standard of care treatment for hepatitis C [110], it became clear that patients of African ancestry had significantly lower cure rates than those of European ancestry during IFN-alpha/RBV treatment. In 2009, two genome-wide association studies discovered IFNL gene polymorphisms as the underlying genetic basis for the different IFN-alpha/RBV treatment responses as well as for different spontaneous clearance rates [111, 112]. This work spurred further investigations on IFNL gene SNPs and their role during HCV infection and treatment.

#### 3.2.1. Role of IFNL Polymorphisms in Antiviral Therapy

**Figure 4:**
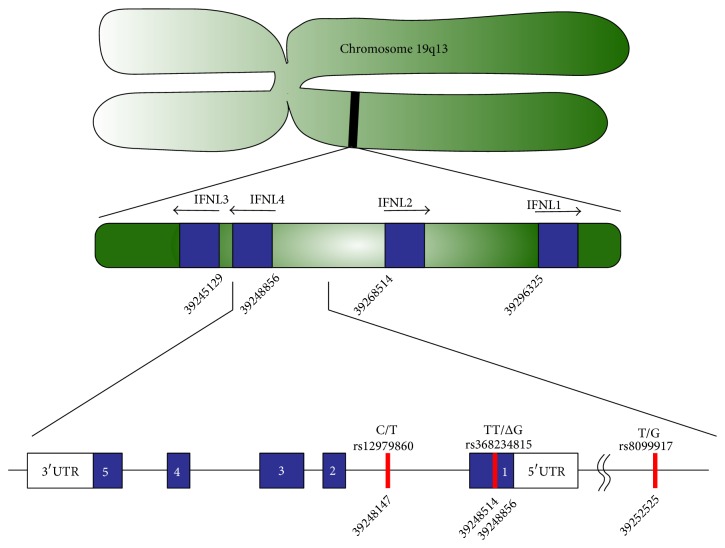
Schematic representation of the IFNL chromosome locus. Depicted is the position and orientation of the genes for IFNL1–4 (blue) on chromosome 19 q13 (green). The numbers below the genes display the nucleotide position of the first exon of each IFNL subtype. IFNL1-2 are located on the forward strand of the chromosome, while IFNL3-4 are located on the reverse strand. The genetic organization of IFNL4 is shown in more detail; it consists of five exons (blue) and 3′/5′ untranslated regions (UTR). Single nucleotide polymorphisms rs12979860, rs368234815, and rs8099917 and their corresponding nucleotide position are indicated (red). Adapted from [38, 39].

Three major SNPs near the IFNL3 and IFNL4 genes correlate with HCV treatment response and are in high linkage disequilibrium [113, 114]. These polymorphisms are rs12979860(C/T) located 3 kb upstream of the IFNL3 gene [111, 112], rs8099917 (T/G) located between the IFNL2 and IFNL3 genes [42, 115], and rs368234815(TT/ΔG) (originally named ss469415590), which creates a frameshift upstream of the IFNL3 gene leading to generation of the new IFNL4 gene product [4, 38, 116]. For all three SNPs the first allelic variant is associated with a higher probability of sustained virological response to IFN-alpha/RBV treatment. The location of these three SNPs on chromosome 19 is schematically depicted in Figure 4.

Treatment response dependency on IFNL polymorphisms was demonstrated for several HCV genotypes and in chronic patients with genotype 4 the IFNL SNPs are the strongest predictors for response known to date [117]. In addition to the three above described SNPs, six additional SNPs in the IFNL locus have been described to strongly associate with sustained virological response after IFN-alpha/RBV treatment (rs8105790, rs11881222, rs8103142, rs28416813, rs4803219, and rs7248668) [115].

How the IFNL SNPs mechanistically influence treatment outcome is mostly unclear. Initially, it was suspected that the SNPs alter the transcriptional regulation of IFNL3 as they are located upstream of the IFNL3 coding sequence, where they could influence transcription factor binding and DNA methylation. However, while some studies detect a correlation of protective IFNL SNPs and higher IFNL expression levels, other fail to do so (see [39] for detailed description). Mechanistically, the rs8099917 SNP has been suggested to influence IFNL3 mRNA stability with the favorable allele being more stable [118]. For the rs368234815(TT/ΔG) SNP the functional impact is best described [4]. The ΔG variant results in the expression of IFNL4, which is a pseudogene in TT carriers. IFNL4 expression is associated with increased ISG levels and this in turn worsens treatment outcome. While this might seem counterintuitive, it is in line with observations that patients with increased pretreatment ISG levels in the liver respond more poorly to IFN-alpha/RBV therapy [119–121]. Thus, it seems at least in the case of IFNL4 that it has an adverse effect during hepatitis C by desensitizing the liver to IFN-alpha/RBV treatment. This has been confirmed in an independent study on the IFNL4 coding SNP rs117648444 [90], which results in a less active IFNL4 variant and consequently in improved treatment response. While one might question the value of these genetic markers in the age of IFN-free DAA treatment with high cure rates, it should be noted that IFNL locus SNPs are also predictive for DAA treatment outcome and moreover influence the DAA response kinetics [122–124]. Genetic markers might therefore allow prediction of treatment duration and consequently reduce costs and exposure time to DAAs.

#### 3.2.2. Association with Spontaneous HCV Clearance

Human polymorphisms in the IFNL locus responsible for improved response to IFN-alpha/RBV treatment are also associated with better spontaneous clearance of HCV.

Allele frequency of the rs12979860 SNP differs between individuals with European or African ancestry with the favorable rs12979860(C) variant predominating in the former population. This finding correlates with better clearance of HCV in European ancestry individuals. The rs368234815(TT/ΔG) SNP similarly predicts HCV clearance rates. However, it is a better predictor than the rs12979860 SNP in African-Americans, while the predictive value of both SNPs is similar in European-Americans [4, 125]. Causative for this difference is the degree of linkage disequilibrium between both SNPs in the two populations [116]. The third SNP with strong predictive value (rs8099917) for the outcome of HCV infection also shows a higher frequency of the protective allele (T) in individuals of European and Asian ancestry as compared to individuals of African ancestry.

In summary, there is a clear link between IFNL gene SNPs and HCV treatment outcome as well as spontaneous clearance. Notably, except for the SNP resulting in IFNL4 expression, the mechanisms causing the association remain elusive and the associated SNPs may not be the true causal variants. Nonetheless, the predictive value of the IFNL SNPs extends beyond hepatitis C as genetic associations with nonalcoholic fatty liver disease, allergy, and infection with cytomegalovirus, human T-lymphotropic virus, hepatitis B virus, HIV, and herpes simplex virus have been suggested (reviewed in [126]). The discovery of IFNL polymorphisms in the context of HCV infection may therefore importantly contribute to the understanding of other hepatic and extrahepatic diseases.

### 3.3. IFNL Therapy

Before the rise of DAAs targeting HCV, IFNLs were considered an attractive alternative to IFN-alpha/RBV treatment for several reasons. The antiviral profile of IFNL resembles the one of IFN-alpha, as both interferons signal via ISGF3. This holds true in primary human hepatocytes [105] as well as in hepatoma cell lines [49]. However the kinetics and magnitude of ISG induction differ between IFN-alpha and IFNL [55, 103, 127]. Another difference between the two IFN types is their tissue specificity caused by the divergent expression pattern of their receptors; in contrast to the IFNL receptor complex, which shows a restricted tissue expression, the IFN-alpha receptor is expressed ubiquitously. Thus compared to IFNL, IFN-alpha acts more systemic, causing more adverse effects, which are often limiting treatment options and compliance.

The overlapping response of IFN-alpha and IFNL signaling on the one hand and the tissue specificity of the IFNLR on the other hand made IFNL promise that the replacement of IFN-alpha by IFNL would yield the same therapy outcome with fewer side effects.

Indeed, clinical studies revealed an improved safety and tolerability profile for PEG-IFNL1a compared to PEG-IFN-alpha [128, 129]. When used in combination with RBV and the DAA Daclatasvir, a 24-week PEG-IFNL1a based treatment does not only show less adverse events, but also leads to a higher sustained virological response than treatment with a PEG-IFN-alpha based regime; 12 weeks after treatment no HCV RNA is detectable in the blood of 88% of patients in the PEG-IFNL1a group compared to only 70.5% of patients in the PEG-IFNL1a group [130]. In a different clinical study PEG-IFNL1a or PEG-IFN-alpha2a were given together with RBV and Telaprevir, but here no noninferiority of PEG-IFNL1a regarding safety, tolerability, and efficacy was observed [131].

IFNL3 has the highest activity among the IFNL types and therefore might be more suitable as therapeutic agent than IFNL1 [55]. Nevertheless, only IFNL1 has been evaluated in clinical trials so far, probably due to the fact that recombinant IFNL3 is difficult to produce. Recently IFNL3 analogs, which allow high yield production and are comparable to IFN-alpha2a in their ability to inhibit HCV replication in Huh-7.5.1 cells, have been designed [132]. However, the licensing of effective DAA paved the way for an IFN-free therapy of CHC, which is becoming the standard of care nowadays. Thus most likely IFNL will not be needed as therapeutic agent for hepatitis C in the future.

While the development of hepatitis C drugs is fortunately an unprecedented success story [133], we still lack specific drugs for other hepatotropic viruses. For instance, hepatitis E virus is sensitive to IFNL in in vitro models [134] and currently treatment options for hepatitis E are limited. Consequently, the mechanistic insights on the interplay of IFNL and HCV might spur important future work on the role and possible therapeutic application of IFNL during infection with other viruses infecting IFNLR expressing tissues, in particular the liver and the lung.

## Figures and Tables

**Table 1:** Amio acid conservation of IFNLs.

	IFNL1	IFNL2	IFNL3	IFNL4
IFNL1	100	72.77	73.82	27.59
IFNL2	72.77	100	95.92	26.89
IFNL3	73.82	95.92	100	28.57
IFNL4	27.59	26.89	28.57	100

Clustal percent identity matrix of IFNL1–4 using MUSCLE showing amino acid identities (%) (protein IDs: Q8IU54, Q8IZJ0, Q8IZI9, and K9M1U5).
